# Phenotypic, genetic, and population structure analysis offer insights into the genetic architecture of root shape in *Beta vulgaris*

**DOI:** 10.1093/hr/uhaf201

**Published:** 2025-07-31

**Authors:** Andrey Vega, Madeline Oravec, Irwin L Goldman

**Affiliations:** Department of Plant and Agroecosystem Sciences, University of Wisconsin Madison, Madison, Wisconsin, United States of America; Horticulture Section, School of Integrative Plant Science, Cornell AgriTech, Cornell University, Geneva, New York, United States of America; Department of Plant and Agroecosystem Sciences, University of Wisconsin Madison, Madison, Wisconsin, United States of America

## Abstract

Root shape is a defining feature of marketability and breeding strategies in the *Beta vulgaris* crop complex encompassing sugar beet, fodder beet, table beet, and Swiss chard. This study leverages the Wisconsin Beta Diversity Panel of 234 accessions to understand the genetic architecture underlying root shape traits, utilizing field trials, genome-wide association, and population structure analyses. High heritability estimates for many root shape traits (*H*^2^ > 0.9) suggest genetic control as the primary determinant of root shape with minimal genotype-by-environment interactions across locations and years. Digital biomass was not correlated with length-width ratio, a key shape descriptor. Key quantitative trait loci (QTL) on Chromosomes 4, 7, and 8 associated with traits such as length, width, and length-to-width ratio, collectively explained up to 55% of phenotypic variance. Several loci co-localize with predicted gene families known to influence organ shape in other plant species. Candidate genes near shape QTL were significantly enriched for microtubule organization and auxin response. Genomic estimated breeding values for shape traits showed high predictive accuracy, particularly for length-to-width ratio. Admixture analyses revealed eight genetic populations, suggesting distinct domestication and breeding histories of crop types in the complex. Swiss chard and wild germplasm showed unique ancestry, while sugar and fodder beet shared genetic proximity. Our analysis identifies candidate loci and molecular markers for root shape, providing resources for molecular breeding strategies in *B. vulgaris*. The findings add to and clarify the current knowledge on root shape inheritance, advancing the genetic improvement of these crops of economic, nutritional, and cultural significance.

## Introduction

The *B. vulgaris* subsp. *vulgaris* crop complex encompasses sugar beet, fodder beet, table beet, and Swiss chard [1]. Sugar, fodder, and table beet are used for their roots, while Swiss chard and some specialty table beet varieties are used as leaf vegetables [2]. All groups primarily share a diploid chromosome number (2*n* = 2*x* = 18), but a few sugar beet varieties are triploid hybrids or tetraploid inbred lines [3]. Since the 2018–2019 crop year, the total value for sugar beet in the USA has exceeded $1 billion USD annually [4]. While table beet, Swiss chard, and fodder beet are economically important in specific regions, they are grown on a comparatively smaller scale worldwide [1]. Nevertheless, table beets have gained popularity as a nutritionally beneficial vegetable [5].

The crop complex traces its ancestry to the wild sea beet (*B. vulgaris* subsp. *maritima*) and forms a genetically compatible and historically intertwined group, offering opportunities for population structure analysis [6–8]. Wild crop relatives such as *B. vulgaris* subsp. *maritima* are cross compatible with cultivated crop types and are routinely used as sources of new alleles in sugar and table beet breeding [9, 10]. These wild relatives do not have swollen roots but do possess ectopic cambia, which have the potential to swell and produce globular, cylindrical, and cone-shaped roots under selection.

*B. vulgaris* domestication began with its wild ancestors being used as leafy vegetables or herbs along the Mediterranean coast, later adopted by Greek and Roman civilizations [2]. The domestication hypothesis suggests that natural mutations and human selection favored the development of enlarged roots and hypocotyls capable of sugar and water storage. Over time, selection transformed the wild populations into a crop with fodder, sugar production, and root vegetable value. However, the molecular mechanisms and inheritance patterns governing the transition to swollen sugar-storing structures remain unresolved.

Root development in plants is a complex process regulated by genetic, hormonal, and environmental factors. Key regulators such as auxins, cytokinins, and gibberellins play central roles in root meristem activity, elongation, and differentiation [11]. Advances in root phenotyping and quantitative genetics have enabled the dissection of root system architecture traits such as root size and shape in root crops like carrots, sweet potato, and cassava [12–17]. Root traits are typically quantitative, controlled by multiple loci with moderate to small effects. Root shape is often described by various traits, with length-to-width ratio and biomass being key indicators of shape and size, respectively.

In *B. vulgaris* the storage organ of sugar and table beets is commonly referred to as a root, but anatomically, only about 90% is true root tissue. The remaining 10%, often called the crown, is a modified hypocotyl [18]. A hallmark of *B. vulgaris* domestication is the diversity in root shapes in table beets including cylindrical, flat, round, spindle, and globose. Selection for various shapes may reflect a desire to achieve particular culinary applications and diverse market classes, improved harvestability, or storability during the postharvest period. The diversity across *B. vulgaris* crops and wild relatives makes them an ideal model for studying the genetic basis of shape. Thus far, genetic studies of shape in roots with horticultural value are limited to a small set of studies in carrot, radish, sweet potato, turnip, and cassava [12–17]. Carrot shape appears to be controlled by polygenic inheritance with additive effects. In radish, potato, and carrot, shape changes have been partly linked to the organ shape regulons OVATE Family Protein–TONNEAU1 Recruiting Motif (OFP-TRM) and IQD (IQ67 domain). Root shape traits in sugar beet have been studied, with one study revealing key loci on chromosome 8, linked to auxin response and nutrient transport [11] and another reporting numerous loci across chromosomes associated with root shape variation [19]. Yet quantitative trait loci (QTL) and their functional roles in other members of the *B. vulgaris* complex and their environmental interactions remain unknown.

Studies of shape in horticultural products started in the 1920s and 1930s when the gene *Ovate* was suggested to control oblong tomato fruits [20]. The gene was mapped and cloned in the 2000s and research has recently advanced to the molecular and cellular level [21, 22]. Shape regulation in plant organs, including fruits, grains, and roots, is influenced by key gene families like the OFP-TRM and IQD plant organ shape regulon [23]. These gene families control developmental pathways and hormonal signaling, with TRMs promoting elongation, OFPs generally repressing it, and IQDs integrating environmental cues to regulate microtubule and cytoskeletal dynamics [24, 25]. In crops like tomato, rice, and radish, these genes have been associated with significant QTL affecting shape [26]. For instance, OFPs and TRMs interact to modulate fruit shape in tomato, potato, cucumber, and peach [24, 27], while OFPs also may play roles in root development, as suggested in carrot and radish [13, 14]. Other genes like *AT-Hook* gene family have also been suggested as important in the transition from wild unswollen carrot roots to swollen storage roots in carrot (*Daucus carota* var. *sativus*) [28]. Despite these advances, the role of these global shape regulatory networks remains unexplored in *B. vulgaris*.

In this study, we assembled a diversity panel and carried out an association analysis with the goals of identifying loci associated with root shape variation in *B. vulgaris*, characterizing the genetic architecture underlying root shape traits, and examining potential overlap between root shape QTL and key plant shape regulatory networks including the OFP-TRM-IQD regulon.

## Results

### Size and shape traits are genetically determined, and size is not correlated with root length-to-width ratio

**Figure 1:**
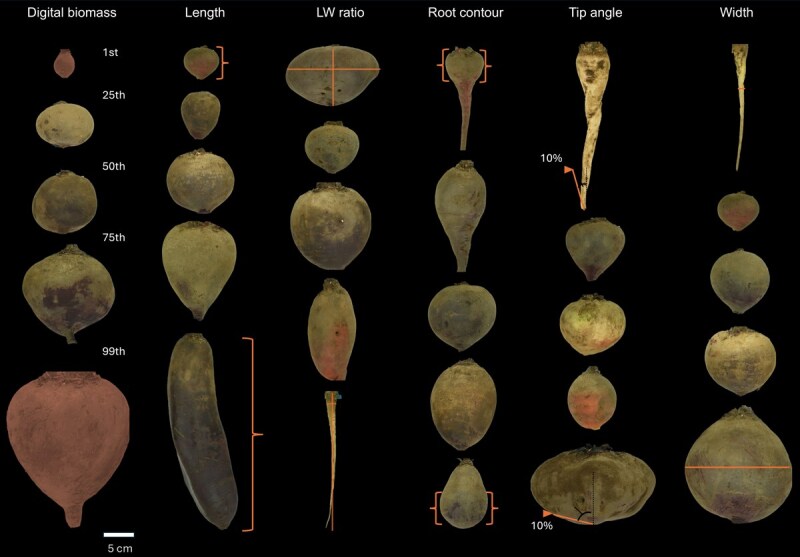
Images of *B. vulgaris* subsp. *vulgaris* accessions and wild relatives illustrating variation across six phenotypic traits: digital biomass, length, length-to-width ratio, root contour, tip angle, and width. Each column represents a trait, with rows showing the 1st, 25th, 50th, 75th, and 99th percentiles of the trait distribution. Percentiles 1 and 99 are annotated for clarity and trait description. Digital biomass is the 2D area of the root in mm^2^; length is the distance from the root crown to the root tip in mm; LW ratio is the log-transformed ratio of root length to maximum width; root contour is derived from principal components (PC) of contour values and tracks the maximum width across the length; tip angle is the interior angle at 10% of length from the midpoint of the tip; width is the diameter at the 50th percentile of length. Annotations in orange are approximate and not drawn to scale.

**Figure 2:**
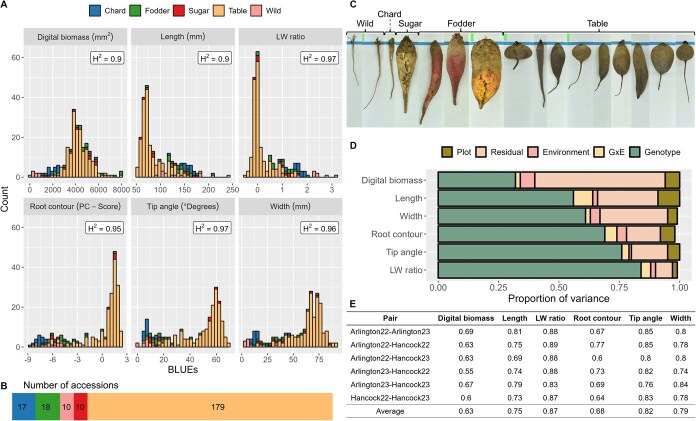
Phenotypic diversity and heritability of root traits in the WBDP. **A** Histograms showing the distribution of six root traits across *B. vulgaris* accessions: digital biomass, length, length-to-width (LW) ratio, root contour (PC score), tip angle, and width. Each bar is color-coded by crop type. Broad-sense heritability (H^2^) values for each trait are indicated. **B** The number of accessions field evaluated for each crop type. **C** Representative root phenotypes of *B. vulgaris* accessions, showing the diversity in root shape and size. **D** Proportion of variance in each trait explained by the sources plot, residual, environment, G × E interaction, and genotype. **E** Pairwise correlations of root traits between pairs of environments and their average.

A total of 234 accessions, representing four crop types and wild relatives with diverse root shapes (Fig. 1), were evaluated using our digital imaging system (Fig. 2B and C and Table S1).

Rainfall, temperature, and relative humidity during the duration of the experiment at both locations and in both years were within the typical range of both metrics for Wisconsin (Table S16). In general, environmental variance was responsible for a small portion of the overall phenotypic variance for all traits (Fig. 2D). Genotype × environment interactions were measured for all traits, but tended to be caused primarily by changes in trait magnitude, rather than rank. These interactions were large only for digital biomass.

Broad-sense heritability estimates were 0.90 or greater, indicating that genotypic phenotypic differences are attributable largely to genotypic differences among accessions (Fig. 2A). Given the dramatic root shape differences among crop types (Figs 1 and 2C), we estimated heritability within each type. The table beet group showed higher heritability for most shape traits compared to the wild relative group (Table S5). In addition, average correlations between pairs of environments indicated similar phenotypic responses across all growing environments (Fig. 2E).

Fodder beet had the largest mean root biomass (5791 mm^2^), while crop wild relatives had the smallest (1015 mm^2^). Table beet was widest on average (66 mm), and crop wild relatives were narrowest (7 mm). Sugar beet had the longest roots (166 mm average), and table beet the shortest (78 mm average). Crop wild relatives had the highest length-to-width ratio, indicating a slim shape, whereas table beet had the lowest, reflecting a rounder form. Taken together, our analysis suggests that the phenotypic diversity in *B. vulgaris* shapes was primarily due to genetic differences rather than non-genetic effects on certain key traits.

Our study found no significant correlation between digital biomass and length-to-width ratio, but significant correlations existed between digital biomass and other root shape parameters. Root shape traits showed strong correlations (0.79–0.93). Tip angle, length-to-width ratio, and root contour primarily defined shape and market class, while digital biomass related primarily to size (Fig. S1).

### Shape traits showed greater stability than biomass across environments

Genotype and G × E (genotype-by-environment) interactions contributed significantly to trait variance, but G × E explained less than 8% of total phenotypic variance (Fig. 2D; Table S6). Genotype was the primary source of variance for all traits except digital biomass, with the strongest genotypic effects on length-to-width ratio, tip angle, and root contour, in that order. Digital biomass had the highest residual variance, highlighting greater environmental influence compared to other traits.

Finlay-Wilkinson regression slopes were used to further study G × E (Table S7; Fig. S2). Overall, we found that accessions with roots <100 mm long, a length-to-width ratio < 1, and a root contour >0 exhibited Finlay–Wilkinson slopes near 1, indicating greater shape stability across environments (Fig. S2B–D). Round, flat, and pear-shaped roots were less sensitive to environmental changes than long, cylindrical, or conical roots. Length, length-to-width ratio, width, and tip angle showed an average environmental correlation above 0.75 (Fig. 2E).

### Population structure is consistent with domestication history and admixture across certain crop types

**Figure 3:**
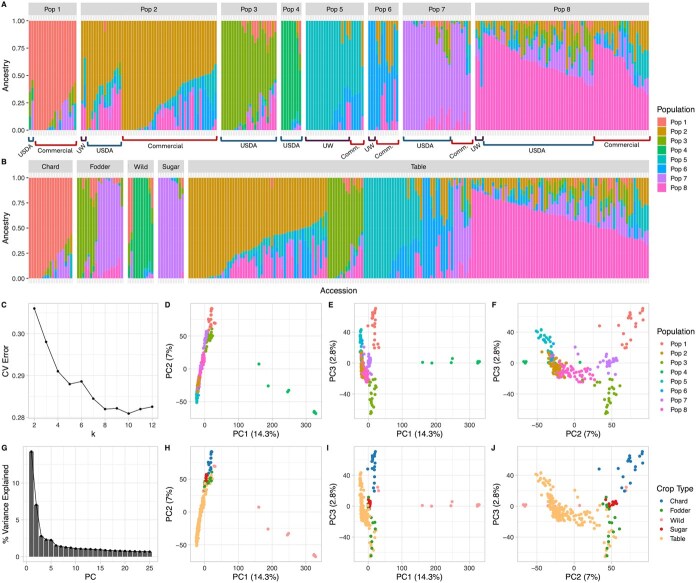
Population structure of 234 *B. vulgaris* accessions. **A-B** Proportion of ancestry attributable to each ADMIXTURE population identified by *k* = 8 sorted by (**A**) population assignment and (**B**) crop type. Each color represents a single population; each column represents one accession; and each colored segment represents the proportion of ancestry (q) contributed from each population. **C** The cross-validation (CV) error from the ADMIXTURE analysis with for population number (*k*) 2 through 12. **D-J** Principal component analysis (PCA) of genotypic data of 234 accessions. PC1, PC2, and PC3 account for 14.3%, 7%, and 2.8% of total variation, respectively. **D-F** Accessions plotted by (**D**) PC1 vs PC2; (**E**) PC1 vs PC3; and (**F**) PC2 vs PC3, with color representing population assignment and shape representing crop type. **G** Percent of variance explained by each PC, 1–25. **H-J** Accessions plotted by (**H**) PC1 vs PC2; (**I**) PC1 vs PC3; and (**J**) PC2 vs PC3, with color and shape representing crop type.

Admixture analysis revealed clear genetic structure among and within crop types, separating accessions into eight populations (Fig. 3; Table 1). Swiss chard accessions and two wild relatives were grouped in Pop 1, suggesting these two wild accessions may be closer progenitors of cultivated types. Sugar beet and Swiss chard accessions primarily traced back to Pops 7 and 1, respectively. Fodder beet split into two groups with one closer to table beet (Pop 3) and the other to sugar beet (Pop 7).

**Table 1:** Pairwise Fst between admixture assigned populations.

	Pop 1	Pop 2	Pop 3	Pop 4	Pop 5	Pop 6	Pop 7	Pop 8
Pop 1	-	0.13	0.07	0.24	0.19	0.17	0.06	0.09
Pop 2	0.13	-	0.11	0.34	0.10	0.08	0.10	0.03
Pop 3	0.07	0.11	-	0.25	0.18	0.16	0.06	0.07
Pop 4	0.24	0.34	0.25	-	0.42	0.41	0.27	0.30
Pop 5	0.19	0.10	0.18	0.42	-	0.12	0.16	0.10
Pop 6	0.17	0.08	0.16	0.41	0.12	-	0.15	0.09
Pop 7	0.06	0.10	0.06	0.27	0.16	0.15	-	0.06
Pop 8	0.09	0.03	0.07	0.30	0.10	0.09	0.06	-

Some table beet accessions showed distinct population assignments, particularly those from the University of Wisconsin (UW) breeding program, which defined Pops 5 and 6. Commercial varieties often showed mixed ancestry, consistent with F1 hybrid development, for example using publicly-available inbred lines from the UW breeding program as their female parent. Pop 3 was comprised entirely of USDA germplasm, which originates from the publicly held National Plant Germplasm System, while Pops 2, 7, and 8 had mixed public and private origins, indicating shared ancestry and breeding history.

**Figure 4:**
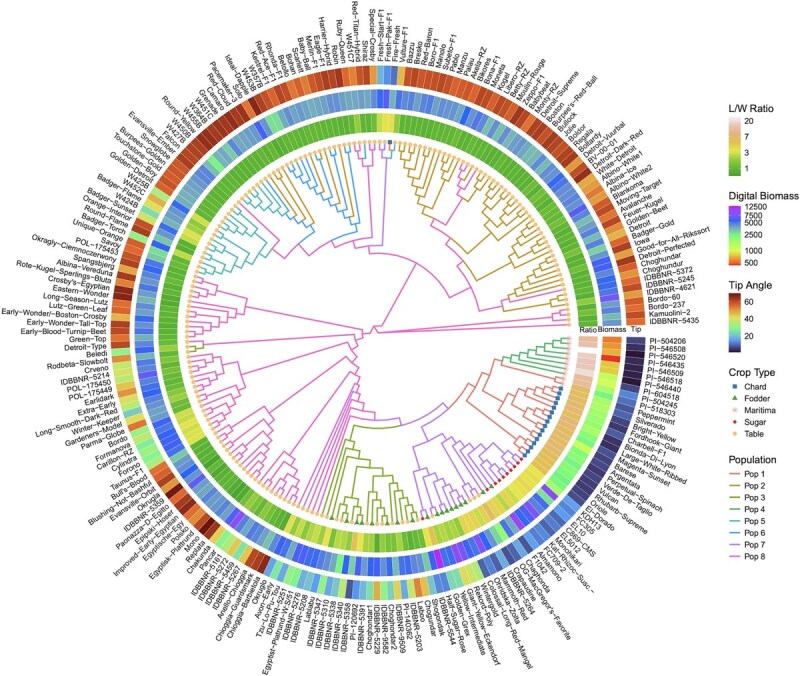
Genetic relationships and shape variation of 234 B. vulgaris accessions. Neighbor-joining tree based on Nei 1972 genetic distance of the 234 *B. vulgaris* accessions. Branches are colored by ADMIXTURE population assignment and tip node point color and shape represent crop type. Surrounding heatmaps show the accession means for three key traits: Length-to-width ratio (inner ring), digital biomass (middle ring), and tip angle (outer ring).

A neighbor-joining tree based on Nei’s genetic distance reflected similar groupings, with wild accessions forming basal clades closest to Swiss chard, fodder, and sugar beet. Table beet was more genetically divergent and spread across multiple clades but also represented a much larger component of the diversity panel. Root shape traits aligned with some genetic clades. For example, high length-to-width ratio and low digital biomass was apparent in the clade representing the wild accessions with long thin roots (Fig. 4).

**Figure 5:**
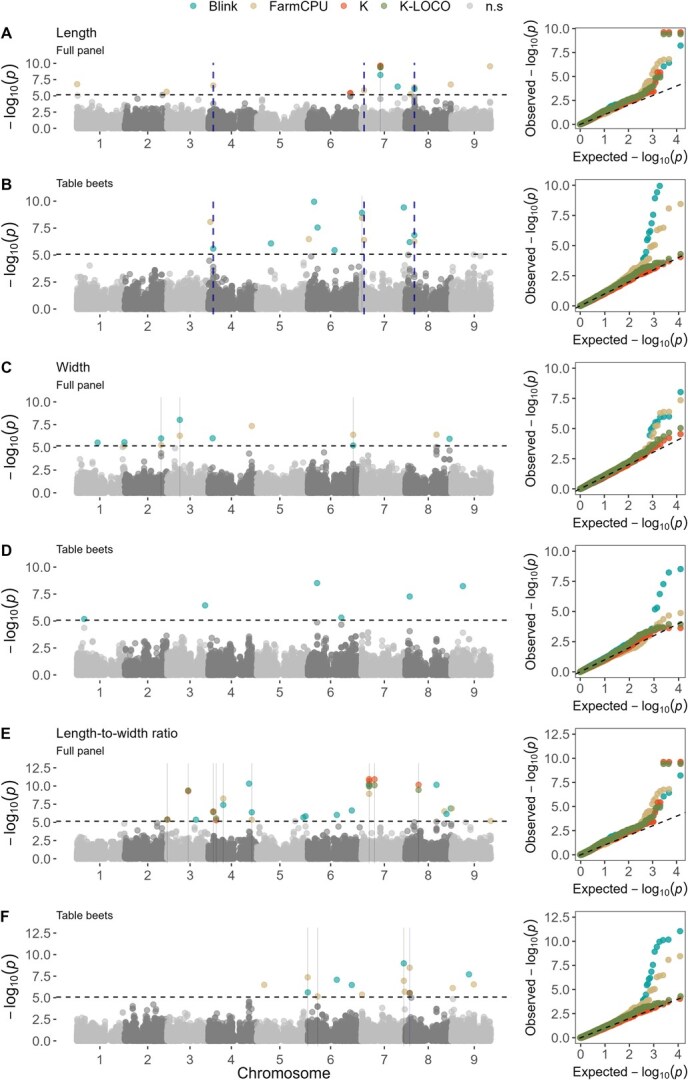
GWAS results for length (mm), width (mm), and log-transformed length-to-width ratio. **A-B** Manhattan and Quantile-Quantile (QQ) plots for the root length in (**A**) the full WBDP panel and in (**B**) a subset of table beets. **C-D** corresponding plots for root width in the (**C**) full panel and (**D**) table beet subset. **E-F** corresponding plots for the log-transformed length-to-width ratio in both the (**E**) full WBDP panel and (**F**) table beet subset. Solid gray vertical lines indicate QTL identified by at least two of the four statistical models tested (Blink, FarmCPU, K, and K-LOCO). A dashed blue vertical line highlights a QTL for length, found in both the full WBDP and table beet subset, located 82 kb apart. The horizontal dashed line represents the Bonferroni corrected significance threshold at α = 0.05. Gray points below the threshold line denote non-significant associations.

Principal component analysis (PCA; Fig. 3D–E) separated wild from cultivated accessions. While crop types showed overlap, PC2 and PC3 differentiated admixture populations, providing evidence of gene flow or shared ancestry between some crop types, especially between fodder and sugar beet. Pairwise Fst values supported these patterns (Tables 1 and 2). Greater variation occurred between genetic populations than crop types, suggesting that traditional crop classifications do not fully capture underlying genetic structure. Pop 4 was the most genetically distinct (Fst 0.24–0.42, Table 1), consistent with wild versus cultivated crop divergence shown in Table 2. Low Fst values (0.04–0.06) between fodder beet and other cultivated types reflect their close genetic relationships, particularly between fodder and sugar beet, consistent with sugar beet’s domestication history.

**Table 2:** Pairwise Fst between crop types.

	Table	Sugar	Fodder	Wild	Chard
Table	-	0.10	0.06	0.23	0.10
Sugar	0.10	-	0.04	0.21	0.07
Fodder	0.06	0.04	-	0.17	0.05
Wild	0.23	0.21	0.17	-	0.18
Chard	0.10	0.07	0.05	0.18	-

### SNP coverage and LD patterns reveal population-specific biases in the WBDP

We achieved sufficient coverage (24 ± 12 reads per individual) across the genome, with no obvious gaps except those near the pericentromeric regions (Fig. S3). The average marker distance was consistent with other reports for the same diversity panel [29–31]. In Table S8, we provide the inter-marker distance statistics per chromosome. A marker correlation of 0.1 extended to 0.12 Mb on average for the full Wisconsin Beta Diversity Panel (WBDP) and 0.54 Mb for the table beet subset (Fig. S4C and D).

We observed a higher proportion of homozygous reference alleles in both the full WBDP panel and the table beet subset indicating a skew toward the reference allele in our accessions (Fig. S4A and B). We hypothesize that the skewness may result from allelic bias, with alternative alleles in haplotypes being unequally amplified and an over representation of table beets in the WBDP.

### Thirty-six significant QTL identified across root shape traits in full WBDP and table beet subset

Across six root shape traits, 36 significant associations were detected across both the full WBDP and the table beet subset, representing 31 unique QTL (Table 3; Figs 5 and S6). Compared to naïve GWAS, all tested models reduced *P*-value inflation (Fig. S5).

**Table 3:** Summary of genome-wide significant marker associations for the full Wisconsin WBDP and a table beet subset

**Panel**	**Trait**	**Chrom**	**Position (bp)**	**Score** ^**a**^	**PVE** ^**a**^	**Model**
Full panel	Length	7	28 827 677	9.08	34	K, K-LOCO, Blink
Full panel	Length	8	13 897 126	6.19	4	FarmCPU, Blink
Full panel	Length	4	7 396 109	6.6	1.4	FarmCPU
Full panel	Length	7	4 116 408	5.8	17.7	FarmCPU
Table beets	Length	7	4 116 408	6.4	0	FarmCPU
Table beets	Length	4	7 396 068	5.6	7.5	Blink
Table beets	Length	7	923 154	8.69	8	FarmCPU, Blink
Table beets	Length	8	13 814 906	6.58	14	FarmCPU, Blink
Full panel	Width	2	55 158 230	5.62	12	FarmCPU, Blink
Full panel	Width	3	19 352 185	7.14	11	FarmCPU, Blink
Full panel	Width	6	68 990 315	5.79	5	FarmCPU, Blink
Full panel	LW ratio	3	340 155	5.4	1	K, K-LOCO
Full panel	LW ratio	3	32 051 258	9.34	3	K, K-LOCO
Full panel	LW ratio	4	7 396 092	6.46	3	K, K-LOCO
Full panel	LW ratio	4	11 783 130	5.42	3	K, K-LOCO
Full panel	LW ratio	4	22 535 303	7.83	5	FarmCPU, Blink
Full panel	LW ratio	4	65 818 310	5.87	30	FarmCPU, Blink
Full panel	LW ratio	7	11 655 715	10.1	11	K, K-LOCO, FarmCPU, Blink
Full panel	LW ratio	7	19 971 511	10.5	0	K, K-LOCO
Full panel	LW ratio	8	20 191 151	9.81	0	K, K-LOCO
Table beets	LW ratio	5	77 160 842	10.9	3	FarmCPU, Blink
Table beets	LW ratio	6	15 009 616	8.94	8	FarmCPU, Blink
Table beets	LW ratio	7	64 394 835	7.97	2	FarmCPU, Blink
Table beets	LW ratio	8	6 726 157	8.28	11	K, K-LOCO, FarmCPU, Blink
Full panel	Root contour	2	39 539 086	6.54	0	K, K-LOCO
Full panel	Root contour	2	60 920 519	7.24	5	K, K-LOCO, FarmCPU
Full panel	Root contour	6	4 307 180	6.48	14	FarmCPU, Blink
Full panel	Root contour	6	50 242 497	5.97	0	K, K-LOCO
Full panel	Root contour	8	44 749 807	6.02	4	K, K-LOCO
Full panel	Root contour	9	28 032 023	6.37	0	K, K-LOCO
Table beets	Root contour	3	50 101 827	7.64	31	FarmCPU, Blink
Full panel	Tip Angle	5	72 933 225	6.43	2	FarmCPU, Blink
Full panel	Tip Angle	9	58 803 335	6.21	15	FarmCPU, Blink
Table beets	Tip Angle	8	6 726 157	9.75	23	FarmCPU, Blink
Full panel	Biomass	3	12 141 936	9.23	19	FarmCPU, Blink
Full panel	Biomass	6	39 837 772	8.49	3	FarmCPU, Blink

a Average LOD score and percent variance explained (PVE) from all significant models. Bonferroni significance threshold 5.1 for the table beets subset and 5.2 for the full panel.

We identified a major QTL for root contour on chromosome (Chr) 3 (50.1 Mb) explaining 31% of the phenotypic variance in the table beet subset (Table 3). This trait is descriptive of a shift in biomass from the crown towards the tip (Fig. 1). Another QTL for root contour on Chr 6 (4.3 Mb) explained 14% of phenotypic variance in the full panel. Significant QTL for tip angle were detected on Chr 5 (72.9 Mb) and 9 (58.8 Mb) in the full panel, and on Chr 8 (6.7 Mb) in the table beet subset, with the table beet QTL explaining 23% of tip angle variance. QLTs associated with digital biomass were only detected in the full WBDP, which were on QTL on Chr 3 (12.1 Mb) and 6 (39.8 Mb); the QTL on Chr 3 explained 19% of phenotypic variation. For length, width, and length-to-width ratio, a total of 19 QTL were identified (Table 3; Fig. 5). The largest effect QTL was identified for length on Chr 7 at 28.8 Mb in the full panel, explaining 34% of root length variance (Table 3  Fig. 5).

### Shared root length QTL on chromosomes 4, 7, and 8 explain notable phenotypic variance in both WBDP and table beet subset

Three QTL were consistently identified for root length on Chr 4, 7, and 8 in both the full panel and the table beet subset (Fig. 5A–B). These loci also overlapped with associations for the correlated trait length-to-width ratio (|*r*| = 0.75; Fig. S1). Together, these loci explained over 20% of the length variation across both the full panel and table beet subset (Table 3).

**Figure 6:**
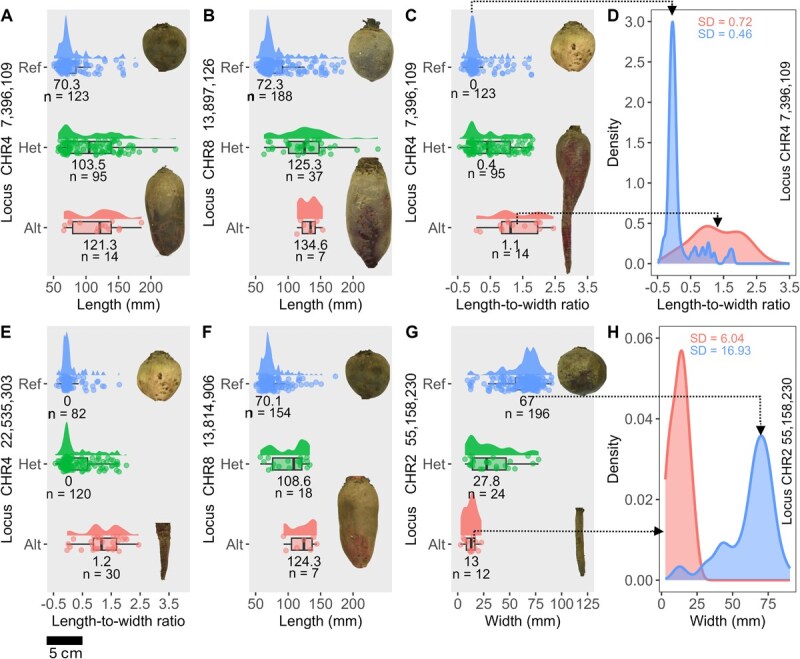
Genotype to phenotype relationship in trait variation of high phenotypic contrast QTL in *B. vulgaris*. **A–C, E–G** Distribution of root length, width, and length-to-width ratio are shown by genotype at seven loci. Genotypes include homozygous reference (Ref), heterozygous (Het), and homozygous alternate (Alt), with sample sizes (n) and median values indicated. Root images represent median phenotypes. **D** and **H** show density plots highlighting allele-specific trait distributions for the QTL on (**D**) Chr 4 at 7.4 Mb and (**H**) Chr 2 at 55.2 Mb.

A significant QTL on Chr 4 (7.4 Mb) was associated with root length in both the full WBDP and table beet subset. The reference allele at this locus was associated with rounder shapes, while, on average, the alternative allele was associated with elongated shapes (Fig. 6A). The median difference between genotypes corresponded to 1.5 standard deviation (SD) units and represented a clear phenotypic difference (Fig. 6A).

The QTL on Chr 7 (4.1 Mb) accounted for 17.7% of root length variation in the full panel (Table 3) and was situated within 1.5 Mb of predicted OFP-family elongation repressors and other members of the OFP-TRM-IQD regulon (Table S10). Individuals carrying the alternative allele showed a median root length increase of 15 mm (0.65 SD; Fig. S8).

Two QTL on Chr 8 at 13.9 and 13.8 Mb were associated with root length variation in the full panel and table beet subset, respectively (Table 3). Given the co-location (only ~82 kb apart) of these two markers, they likely represent the same locus. The QTL at 13.8 Mb explained 14% of the root length variation in the table beet subset and distinguished between round and elongated shapes (Table 3; Fig. 6F). For both these QTL, the reference allele was associated with round shapes, while alternative alleles were associated with elongated roots with differences up to 1.77 SD units between genotypic classes.

### QTL for width and length-to-width ratio distinguish between swollen storage root and unexpanded taproots

We identified three QTL associated with root width on Chr 2, 3, and 6 in the full WBDP, collectively explaining 28% of the phenotypic variance (Table 3; Fig. 5C and D). The largest effect QTL for root width was identified on Chr 2 at 55.2 Mb (Table 3). Individuals with homozygous alternative alleles at this locus had a median width of 13 mm, while those with homozygous reference alleles had a median width of 67 mm, representing a difference of 2.5 SD units (Fig. 6G). This QTL effectively distinguished phenotypes of cultivated table beets from wild crop relatives with unswollen or unexpanded taproots (Fig. 6H; Table S13). The two additional width QTL on Chr 3 and 6 (Table 3) showed small additive effects, with phenotypic differences between 0.5 and 1.5 SD units for individuals with two copies of the reference or alternative alleles, respectively (Fig. S8).

Nine QTL for length-to-width ratio collectively explained 55% of the phenotypic variance in the full WBDP (Table 3). Meanwhile, four QTL were identified for this trait in the table beet subset, explaining 25% of the variance (Table 3; Fig. 5E and F). This is in line with the expectation of greater shape diversity and more loci contributing to shape variation in the full panel compared to the table beet subset.

Overall, width and length-to-width ratio QTL distinguished between the swollen round table beets and the minimally developed wild type taproots of wild crop relatives and Swiss chard (Fig. 6E, G, and  H) or between round-rooted table beets and other cylindrical or elongated shapes (Fig. 6C and D).

### Gene ontology analysis revealed enrichment for response to auxin and microtubule dynamics in candidate genes

Candidate genes in LD with all significant root shape-associated QTL were identified and are listed in Table S9. Gene ontology (GO) enrichment analysis of target candidate genes revealed significant overrepresentation of genes associated with microtubule nucleation, cytoplasmic microtubule organization, and response to auxin (Table 4). No significant enrichment was found for GO terms in the cellular components or molecular function domains.

**Table 4:** Top GO terms for BPs enriched among candidate genes

GO.ID	Term	P-value^a^
GO:0009733	response to auxin	0.04
GO:0007020	microtubule nucleation	0.04
GO:0031122	cytoplasmic microtubule organization	0.04
GO:0006396	RNA processing	0.12
GO:0007018	microtubule-based movement	0.15

a Fisher’s exact test for GO term enrichment, as implemented in the topGO package.

### Root shape traits show higher genomic predictability compared to biomass

Genomic estimated breeding values (GEBVs) of root shape traits, including width, length-to-width ratio, tip angle, root contour, and length, showed high predictive accuracy (*r* > 0.88) and coefficients of determination (*r*^2^) above 0.78 (Table 5). Among these, length-to-width ratio exhibited the strongest predictive performance, with an r of 0.95 and *r*^2^ of 0.90. These results suggest reliable in prediction of root shapes from genomic data. In contrast, digital biomass showed lower predictive accuracy, with an *r* of 0.57 and *r*^2^ of 0.32.

**Table 5:** Accuracy and Coefficient of Determination for GEBVs across six root shape traits of *B. vulgaris* in WBDP

Trait	Accuracy (r)	Coefficient of Determination (r^2^)^a^
Digital biomass	0.57	0.32
Width	0.88	0.78
Length-to-width ratio	0.95	0.90
Tip angle	0.92	0.85
Root contour	0.90	0.82
Length	0.89	0.78

a Coefficient of determination is estimated as accuracy squared and represents the proportion of phenotypic variance explained by GEBVs.

## Discussion

Our results indicate that many of the root shape parameters are highly heritable in *B. vulgaris*. High estimates of heritability in image-based shapes have been also recently reported in apple, potato, and sweet potato [15, 32, 33]. Length-to-width ratio has been reported as one of the best descriptors of shape for roots of horticultural value, fleshy fruit, and even grains [34]. Our findings confirm that length-to-width ratio, which effectively captures complex root geometry in a single trait, is genetically independent of size traits such as digital biomass and is a good descriptor of root shape.

The observed genetic separation and admixture among and within crop types align with the known domestication history in *B. vulgaris*, illustrating how historical selection and breeding practices have shaped the genetic structure of the crop complex. The distinct genetic clustering of Swiss chard and wild germplasm supports the notion that Swiss chard represents one of the earliest domesticated forms of *B. vulgaris*, selected for leafy, rather than root traits [35]. The nesting of sugar beet accessions among fodder beets, with close genetic relatedness to both fodder and chard reflects the direct derivation of sugar beet from the hybridization of fodder beet and chard, as breeders identified and selected for sucrose content [36]. Genetic divergence observed between the wild accessions (Pop 4) and the domesticated root crops offers a potential valuable reservoir for introducing novel allelic variation into cultivated germplasm. Genetic divergence of table beet populations and their division into multiple clades reflects intense selection on specific root shapes, sizes, and pigmentation patterns, and the siloed nature of both public and private breeding programs. Similar genetic isolation of table beet germplasm has been observed in other studies [30, 37]. Admixture within populations indicates some gene flow between groups, yet it is clear there is untapped potential for allelic variation and trait introgression across groups. The observed population structure, coupled with shape variation, mirrors the known selection of *B. vulgaris* into distinct crop types and indicates that breeding efforts have remained largely within crop types. Together, these results highlight that considering population structure and admixture patterns in breeding can help effectively incorporate underutilized sources of genetic diversity across the *B. vulgaris* crop complex.

Although significant, the G × E interaction accounted for less than 8% of the total variation (Table S6). Root shape was less affected by G × E than root size, with round shapes showing less variation compared to cylindrical or elongated types. Our findings highlight the greater influence of genotype over G × E interactions in determining root shape in *B. vulgaris*, consistent with previous studies on horticultural roots [13].

### Root shape in *B. vulgaris* is a highly polygenic trait controlled by additive allelic effects.

Multiple loci contributed to root shape, aligning with previous studies on horticultural roots like carrots, potatoes, and turnips [13, 15, 16]. Most QTL showed additive inheritance (Figs 6 and S8) and were widely distributed across the genome, making them strong candidates for genomic selection. We identified five pleiotropic QTL, associated with more than one trait. One example is the locus on Chr 4 at 7.4 Mb, which was associated with both length (Fig. 6A) and length-to-width ratio (Fig. 6C). Another was a QTL on Chr 8 at 6.7 Mb linked to length-to-width ratio and tip angle, and explaining 11.4% and 23% of phenotypic variance, respectively.

Despite the highly polygenic nature of root shape, we found a root width QTL on Chr 2 at 55.2 Mb that reliably distinguished between table beet and the elongated shapes of crop wild relatives, Swiss chard, and sugar beet (Table S13; Fig. 6G and H). This marker could be used in marker-assisted or genomic selection for inter-crop type crosses, to select and preserve desirable root shape while introgressing other beneficial traits from diverse germplasm in *B. vulgaris*. This locus is a strong candidate for further validation and studies on the dynamics of root shape and the domestication history of *B. vulgaris*.

We identified a biomass QTL on Chr 6 at 39.8 Mb, located just 1.7 Mb from a significant SNP associated with biomass reported by an independent study using 188 sugar beet accessions [19]. That study reported numerous significant associations for root morphological traits, supporting our hypothesis that shape and size are highly polygenic.

Genomic estimated breeding values (GEBVs) represent the genetic potential of an individual for a given trait. Reported values can be used to guide parent selection for each trait in breeding programs of *B. vulgaris* (Table S12). Traits associated with root shape traits displayed high GEBV predictive accuracy and explanatory power, while genomic predictive performance was lower for digital biomass. The reduced predictive performance for biomass may result from greater environmental influence on this trait (Fig. 2). These results indicate that future genomic selection models may perform very well for predicting root shape traits and acceptably for size traits (Table 5). The highest *r*^2^ was obtained for length-to-width ratio, supporting it as one of the best descriptors of root shape and a promising molecular selection target for breeding purposes (Table 5).

### Root shape QTL co-localize with genes associated with the OFP-TRM-IQD regulon, auxin signaling, and AT-hook motifs

Several root shape QTL co-localized within 500 kb of genes from the OFP-TRM-IQD plant shape regulon, including two table beet-specific QTL on Chr 7, one near an OFP gene and the other near a TRM gene (Fig. S9). These findings highlight the proximal and distal ends of Chr 7 as hotspots where OFP-TRM and IQD regulon members are in close genetic or physical proximity to QTL for root shape traits in *B. vulgaris* [38].

The OFP-TRM-IQD regulon, a key regulator of shape in fruit, grains, and tubers, had previously been examined for its relationship to root shape in both radish and carrot [13, 14]. Using bioinformatics and conserved motifs, we examined overlaps between this regulon and identified QTL in *B. vulgaris*. In the W357B genome, we identified 13 OFPs, 18 TRMs, and 26 IQDs with sequence homology to *A. thaliana* TAIR10 (Fig. S9; Table S10) [39, 40].

We found that the TRM family of genes lacks detailed functional annotations in *B. vulgaris* and other root crops [13]. While studies in *A. thaliana* and wheat suggest TRMs are part of a gene complex stabilizing microtubule orientation during root cell division, most TRMs in *B. vulgaris* are annotated as domains of unknown function (DUF4378/DUF3741) [41, 42]. Our phylogenetic analysis showed that peptide sequences of *B. vulgaris* TRMs aligned with several of the eight TRM groups in *A. thaliana* and their sequences contain conserved TRM motifs [41] (Fig. S7**;**  Table S10).

We identified seven QTL within 500 kb and two in LD with members of the OFP-TRM-IQD regulon (Fig. S9). For instance, the root length QTL on Chr 7 at 923 kb is in LD with OFP1 (Bevul.7G008500.1, Table S9) and the QTL on Chr 7 at 64.4 Mb is in LD with a putative TRM gene (Bevul.7G206300.1, Table S10), known elongation repressors and promoters, respectively, in model species [34]. Recently, a GWAS in apple identified OFP family genes as candidates that differentiated flat-shape fruit from round or elongated fruit types [32].

Auxin and gibberellin signaling pathways are recognized as key regulators of growth and organ shaping, including sugar beet and apple [19, 32]. We found that the QTL on Chr 4 at 65.8 Mb, explaining 29% of length-to-width ratio variation, is just 40 kb from an auxin response gene. This proximity and the high proportion of phenotypic variance explained suggests that variation within or near this locus may influence auxin-responsive gene expression, contributing to root developmental variation.

In our study, we found significant GO enrichment for candidate genes involved in microtubule organization and auxin-mediated signaling, implicating cytoskeletal remodeling and hormone-regulated cell expansion as key contributors to root morphogenesis (Table 5; Fig. S10). These findings align with broader evidence across vegetable crops, where shape variation is often driven by allelic differences in genes regulating cell division and elongation, as well as hormone transport and signaling pathways [43].

A marker on carrot chromosome 2 linked to an *AT-hook nuclear motif* gene has been associated with root tissue patterning and the distinction between wild type and domesticated swollen storage roots [28]. In our study, we also identified a predicted *B. vulgaris AT-hook family gene* 6 kb from and in LD with a QTL for length-to-width ratio on Chr 5 (77.2 Mb) in the table beet subset (Table S9). This may suggest a conserved role for A*T-*hook genes in root shape modulation across species [28, 44].

## Conclusion

Fresh market interest in crops of the *B. vulgaris* complex, especially table beets have increased demand for cultivars with novel and appealing root shapes. Shape modifications in these crops have the potential to increase consumer interest through the development of novel and appealing shapes. However, breeding for new shapes is challenging without knowledge of the shape inheritance patterns. Our findings reveal key QTL that control important root shape traits, particularly on Chr 2 (width at 55.2 Mb), Chr 4 (length and length-to-width ratio at 7.4 Mb), and Chr 8 (length and tip angle at 6.7 Mb). These loci are strong candidates for marker-assisted selection to enable broader genetic recombination while maintaining desirable phenotypes. We also report that root shape inheritance in *B. vulgaris* is polygenic and additive, like other roots of horticultural value such as carrots.

In addition, the high predictive accuracy of GEBVs (*r* > 0.88) across most traits supports the implementation of genomic selection, including for traits with moderate heritability like tip angle and root contour. Ongoing fine-mapping efforts using bi-parental populations aim to validate these QTL and clarify their functional roles.

## Materials and methods

### Plant materials and experimental design

We used 234 accessions of *B. vulgaris* L. subsp. *vulgaris*, including representatives of Swiss chard, fodder beet, sugar beet, table beet, and wild relatives from the WBDP (Tables S1 and S2) for this study [29–31]. We conducted field trials at the Hancock and Arlington Agricultural Research Stations (ARS), University of Wisconsin-Madison, in the years 2022 and 2023.

The Hancock site features Plainfield sand and Pearl and Friendship loamy sand soils (Typic Udipsamments and Arenic Oxyaquic Hapludalfs) and was consistently irrigated during the growing season. Arlington, which relies on natural rainfall, has Plano silt loam soil (Typic Argiudolls). At Arlington, experiments were sown on 25 May 2022 and 24 May 2023, and harvested on 15 August 2022 and 22 August 2023. At Hancock, experiments were sown on 17 May 2022 and 25 May 2023, with harvests on 17 August 2022 and 15 August 2023.

Arlington received 31.8 cm (2022) and 22.0 cm (2023) of rainfall, while Hancock received 33.6 cm (2022) and 20.8 cm (2023). Average relative humidity was 71.6% (2022) and 65.5% (2023) at Arlington, and 72.7% (2022) and 67.9% (2023) at Hancock. Average temperatures at 2 m height were 21.4°C (2022) and 21.5°C (2023) at Arlington, and 20.1°C (2022) and 20.8°C (2023) at Hancock (Table S16).

We planted the full WBDP in Arlington and a subset of the panel in Hancock both years (Table S1). Due to the low seed availability of 109 accessions, we partially replicated the panel. At Arlington, we planted 109 accessions in short rows, with the accession of interest in the middle 0.6-m section of a 1.8-m row and table beet cultivars ‘Ruby Queen’ or ‘Bull’s Blood’ were planted in the outer regions of those rows (0.6 m on either side of the accession). The other 125 accessions were planted in full 1.8-m rows (long rows) at both locations for a total of 234 accessions in Arlington and 125 in Hancock (Fig. S11). Long rows were planted using a push planter (Planet Jr, Cole Planter Company, Albany, GA) and short rows were planted by hand. Across experiments, we used a randomized complete block design (RCBD) with one experimental unit replication (row) per block in each of three blocks. Planting and harvesting dates, soil type, seeding rate, as well as experimental details, are provided in Table S3. We harvested six to ten roots per row, or all available roots if fewer than six were present, from the middle 0.6-m section of all rows, avoiding broken or overgrown roots. Harvested roots were stored at 5°C. Across all locations, years, blocks, and accessions, we phenotyped a total of 10 845 roots (Table S11).

### Phenotyping

Phenotyping was carried out through digital imaging and analysis [45]. The imaging system was optimized and validated against manual measurements for *B. vulgaris* (Fig. S12). Prior to imaging, soil and root hairs were scrubbed from the roots using a towel or brush and root tips and lateral branches were removed. Experimental units more than 3.0 standard deviation units from the mean were inspected and removed if they showed morphological abnormalities or did not match the expected shape of their group (see Supplementary Materials, code 01-BLUEs).

The traits were defined as follows: Digital biomass was measured as the 2D mask area (mm^2^). Length (mm) was the distance from the root crown center to the root tip. Maximum width (mm), the widest diameter of the root, was used to calculate the length-to-width ratio, a unitless trait (mm/mm) derived by dividing length by maximum width. Raw length-to-width ratios were log-transformed using the *base::log* function in R to meet GWAS assumptions, with transformed data presented throughout the manuscript and untransformed data available in Table S11. Width (mm) was recorded as the root diameter at 50% of its length. Tip angle was defined as the interior angle formed by lines connecting the root tip center to contour points located 10% of the root length from the bottom. Root contour, capturing the swollen region’s relative position along the root, was derived via principal component analysis on contour values. Representative roots from the 1st, 25th, 50th, and 99th percentiles of the distribution are shown in Fig. 1. Size-independent variation in PCA-derived root contour was normalized as previously described [45].

### Genotyping

At least 2 grams of leaf tissue were collected from 13 ± 4 plants from each accession (Table S4), freeze-dried, and stored at −80°C. The University of Wisconsin-Madison Biotechnology Center conducted DNA extraction from less than 50 micrograms of freeze-dried pooled tissue, followed by library preparation, and genotyping-by-sequencing. The DNA was digested with the restriction endonucleases *NsiI* and *BfaI*. DNA was ligated to barcoded Illumina adapters for sequencing on a NovaSeq6000 sequencer (Illumina Inc., San Diego, CA), with a read length of 2 × 250 bp. Reads were preprocessed by trimming adapters, selecting read size, and removing low-quality reads (Phred score < 20), all using Skewer [46]. On average, 3.56 million reads were obtained per sample across all 234 samples. Variant discovery was conducted by the Bioinformatics Resource Core (https://bioinformatics.biotech.wisc.edu/) using Tassel GBS Version 2 [47]. Read alignment was performed using Bowtie2 [48] and the table beet reference genome *B. vulgaris* subsp. *vulgaris* ‘W357B’ [49].

Marker filtering was performed using bcftools [50]. Only biallelic, polymorphic markers with a read depth in the 90th percentile (≥10), a minimum genotype quality (GQ) of ≥20, and at least five individuals per homozygous genotype were retained. Markers with a genotype frequency > 1–5/*N*, where *N* = number of accessions, were removed resulting in a VCF file with 7263 markers for the full panel. We then divided the diversity panel to only encompass accessions categorized as table beets (Table S1) and performed identical filtering procedures resulting in a VCF file with 6001 SNP markers.

### Statistical analysis and data management

Phenotypic values for GWAS and other analyses were obtained by modeling ${g}_i$ as a fixed effect, with other parameters treated as random [51]. Best linear unbiased estimates (BLUEs) and variance components for each trait were estimated using the R package *lme4* and the following linear model:


$$ {y}_{ij kl}=\mu +{g}_i+{e}_j+g\times{e}_{ij}+{b}_{k(j)}+{p}_{l(jk)}+{\epsilon}_{ij kl} $$

where ${y}_{ijkl}$ is the observation from a root, $\mu$ is the intercept, ${g}_i\sim N\left(0,{\sigma}_g^2\right)$ is fixed main effect of genotype, ${e}_j\sim N\left(0,{\sigma}_e^2\right)$ is the random main effect of the environment defined as the location-year combination, $g\times{e}_{ij}\sim N\left(0,{\sigma}_{ge}^2\right)$ is the random two-way genotype by environment (G × E) interaction effect, ${b}_{k(j)}\sim N\left(0,{\sigma}_b^2\right)$ is the random main effect of block nested within environment, ${p}_{l(jk)}\sim N\left(0,{\sigma}_{plot}^2\right)$ is the random main effect of the plot that tracks the variation of roots within the plots, and ${\epsilon}_{ijkl}\sim N\left(0,{\sigma}_{\epsilon}^2\right)$ is the random error or residual effect associated with the observation ${y}_{ijkl}$.

Broad-sense heritability (*H*^2^) was estimated using the average pairwise prediction error variance (PEV) of ${\sigma}_g^2$ effects derived from BLUPs, calculated with the R function *Agriutilities::h_cullis* [52] as:


$$ {H}^2=1-\frac{\mathrm{PEV}}{\sigma_g^2} $$

We report heritability at the population level (Fig. 2) and by crop type (Table S5). Trait correlations were estimated in R using *GGally::ggpairs* and other correlation analyses were performed using *stats::cor.*

### Genotype × environment interactions

For genotype × environment (G × E) interaction analysis, we selected 104 accessions with complete replication across all environments. We estimated a phenotypic BLUE for each accession within each environment by refitting the model and excluding the ${e}_j$ and $g\times{e}_{ij}$ terms. We calculated the Finlay–Wilkinson slope of each accession’s phenotypic value relative to the average value of all accessions within each environment [53].

### Population structure analysis

Markers were pruned based on linkage disequilibrium using PLINK v2 [54] with a 50-kb sliding window every 5 kb with an *r*^2^ cutoff of 0.02, resulting in 6966 independent markers representing the full panel. ADMIXTURE (version 1.3.0) was used to estimate population structure and ancestry for *k* values ranging from 1 to 12 [55]. The optimal number of populations (*k* = 8) was selected based on the minimum cross-validation error. PCA was performed using *prcomp* function to visualize genetic variation among individuals. A Nei (1972) genetic distance matrix was calculated using the function *ad4::dist.prop*, method 4 (version 1.7-22) [56]. A neighbor-joining tree was constructed using the *ape::nj* function (version 5.6-2) [57] and visualized with associated phenotypic data using the *ggtree* package (version 3.4.0) [58, 59]. Pairwise Fst values were calculated to assess genetic differentiation between crop types and populations using the *hierfstat*:*:pairwise.neifst* function (version 0.5-11) in R [60].

### GWAS analysis and LD

BLUEs were used as phenotypic values for association mapping. GWAS was conducted using the full WBDP and a subset of table beet accessions in GWASpoly and GAPIT V3 [61, 62]. Population structure was controlled using a kinship matrix (K) or the K-LOCO method in GWASpoly and a default Van Raden K matrix in GAPIT. Four models were applied: a Mixed Linear Model [(1) with K and (2) with K-LOCO] and the additive model in GWASpoly, and (3) FarmCPU and (4) Blink in GAPIT.

For all models, a Bonferroni correction at α = 0.05 was applied. *P*-value inflation (*Lambda*) was assessed using the linear regression coefficient of observed vs expected –log10 *P*-values and QQ plots. To minimize Type I error, markers were deemed significant if identified by at least two models or in both the full panel and table beet subset. The first three principal components of the genotypic matrix were used as additional controls (Fig. S5).

Two accessions were excluded from GWAS and some phenotypic analyses due to low-quality data (Table S4). Genome-wide LD was estimated using *geneticMapR* [63] at an *r*^2^ threshold of 0.1 determining LD block size. The percent variance explained (PVE) for each QTL was calculated using outputs from GWASpoly and GAPIT.

### Candidate gene search and GO

Gene annotations from the sugar beet reference genome (*B. vulgaris* EL10.2) were transferred to the table beet reference genome (W357B) using sequence homology and the Liftoff package [49, 64]. The W357B liftoff annotation was queried with Integrated Genomics Viewer, and Phytozome v13 (https://phytozome-next.jgi.doe.gov/) was used to identify candidate genes based on annotated or predicted functions.

We focused on homologous genes in the OFP, TRM, and IQD families to assess colocalization of QTL with predicted proteins. Homologs were identified using conserved motifs and the BLAST tool in Phytozome v13. For IQD genes, the IQ67 consensus motif was used [65]; For the OFP family in, we used the OVATE consensus motif from *Solanum lycopersicum* and *Arabidopsis thaliana* [14, 24]. For the TRM family, six motifs were first identified using The MEME Suite (https://meme-suite.org/meme/index.html), and all 34 *A. thaliana* TAIR10 TRM gene members [66]. To further characterize poorly annotated genes near QTL, multiple sequence alignments were performed with Clustal Omega, incorporating known shape-regulating genes from *A. thaliana*. Motif alignments with peptide sequences from both species were conducted using the MAST tool in The MEME Suite (Table S16) [67].

GO enrichment analysis was performed using the topGO package in R (v.2.52.0). Candidate genes located within 500 kb of GWAS-identified QTL (Table S14) were classified as either target (1) or background (0) and provided as a binary named vector, 16 target candidate genes were selected out of the 423 total candidate genes based on expected potential involvement in shape regulation. Gene-to-GO mappings were curated and formatted as required by the *annFUN.gene2GO* function. Enrichment was assessed for biological process (BP) terms using Fisher’s exact test with the ‘weight01’ algorithm, which accounts for the GO topology to reduce false positives. The top five enriched terms were visualized using ggplot2.

### Genomic Estimated Breeding Values

We calculated Genomic Estimated Breeding Values (GEBVs) for all traits using the *rrBLUP* in R [68]. GEBVs were computed for individuals using 100% of the data as the product of the genotype matrix and marker effects. We split the data into an 80% training set and a 20% testing set for cross-validation to estimate accuracy. Accuracy (*r*) was calculated as the Pearson correlation between GEBVs and observed phenotypes in the testing set. Coefficient of determination was estimated as accuracy squared (*r*^2^).

## Supplementary Material

Web_Material_uhaf201

## Data Availability

Supplementary Figures, Tables, code, and files are all available locally in Zenodo (https://doi.org/10.5281/zenodo.15482778).
