# Supplementary material for: Phenotypic, genetic, and population structure analysis offer insights into the genetic architecture of root shape in Beta vulgaris
Source: Hortic Res. 2025 Jul 31;12(11):uhaf201. doi: 10.1093/hr/uhaf201 (PMC12574335; doi:10.1093/hr/uhaf201)
Supplement: Web_Material_uhaf201 [file web_material_uhaf201.zip › Supplemental_Figures_2025_06_26.pdf]

Supplementary Figures

Phenotypic, genetic, and population structure analysis offer insights into the genetic architecture of root shape in *Beta vulgaris*

A. Vega, M. Oravec, I. Goldman. 2025.

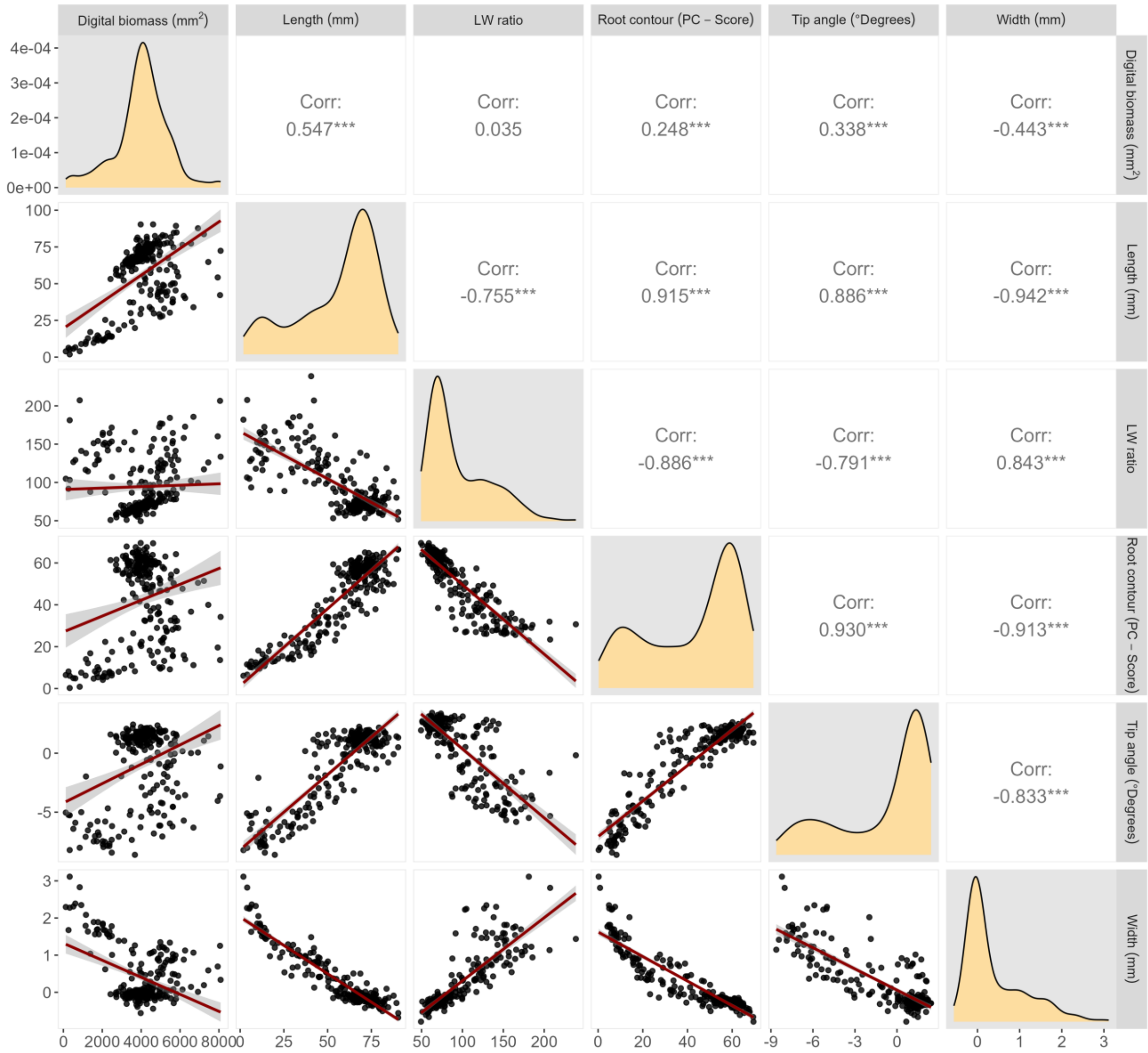

Figure S1. Matrix of pairwise scatterplots and Pearson correlation coefficients of BLUEs for six shape traits: digital biomass (mm^2), length (mm), length-to-width ratio (LW ratio), root contour [Principal Component (PC) -score], tip angle (degrees), and width at the 50th percentile of length (mm). The diagonal show BLUEs distributions for each trait as density histograms. The off-diagonal lower panels show the relationship between pairs of traits each fitted with a linear regression red

line and a 95% confidence interval. The upper off-diagonal panels show the correlation coefficients between traits. Asterisks indicate significance levels: ns, ***p<0.001. LW ratio is log-transformed.

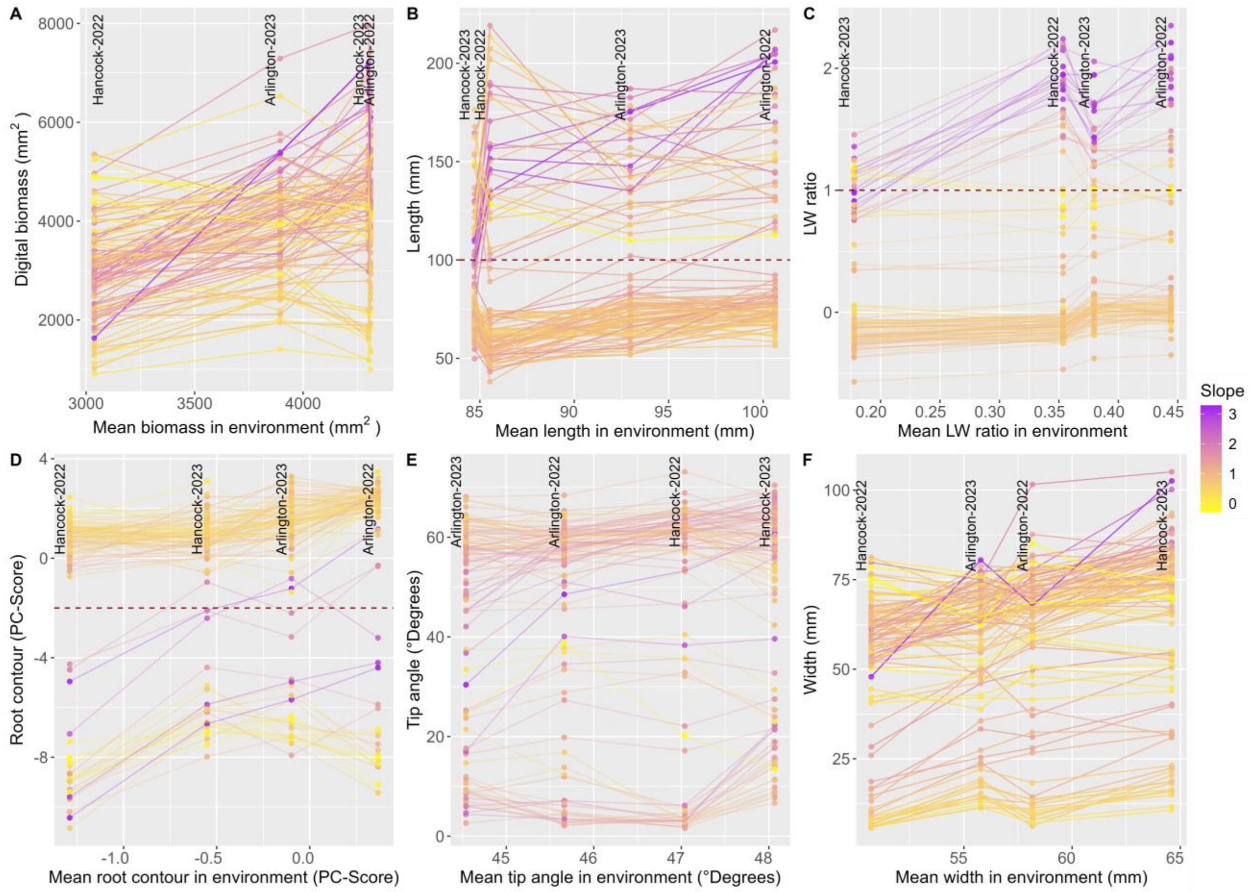

Figure S2. Genotype by environment interactions for a range of root traits from roots grown in multiple years and environments, with the x-axis showing the mean trait values of all accessions for each environment, and individual accession trait values represented as points. Lines illustrate each accession's slope relative to the mean trait value across all accessions in that environment. The y-axis represents trait values for six shape traits. **A** Digital biomass, **B** Length, **C** Length-to-width ratio (LW ratio), **D** Root contour, **E** Tip angle, **F** Width at the 50th percentile of length. Positive slopes indicate an increase in an accession's mean trait value relative to the mean trait value of all accessions in an environment, while negative slopes indicate a decrease in accession's mean trait value relative to the mean trait value of all accessions. The dashed red lines in **B**, **C** and **D**, represent visual thresholds that divide environmentally responsive from stable accessions.

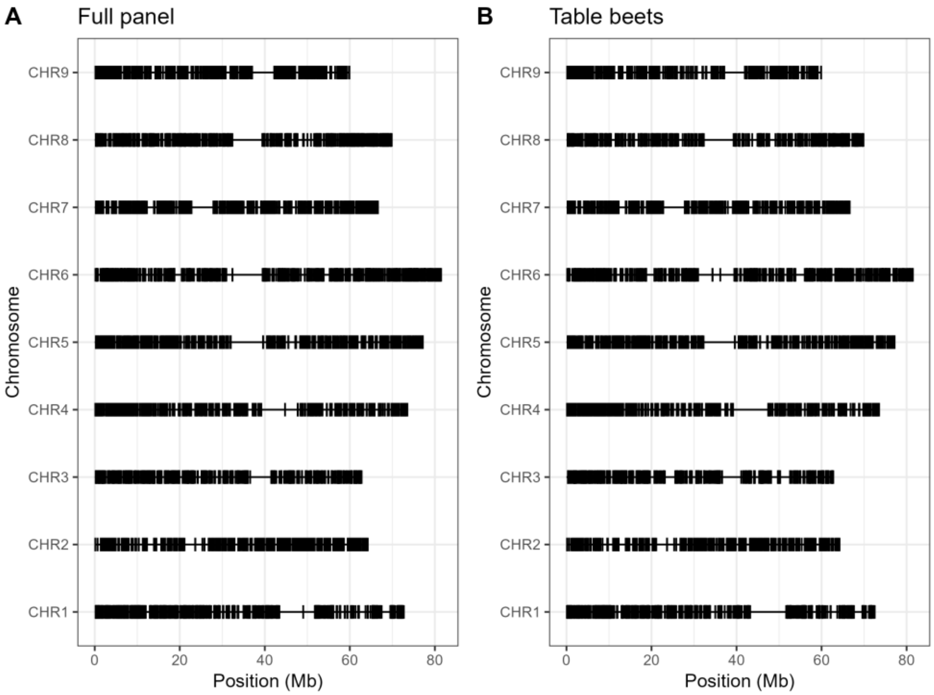

Figure S3. Genome-wide marker coverage using 7263 and 6001 SNPs for the **A** full Wisconsin Beta Diversity Panel (WBDP) and **B** table beets only subset.

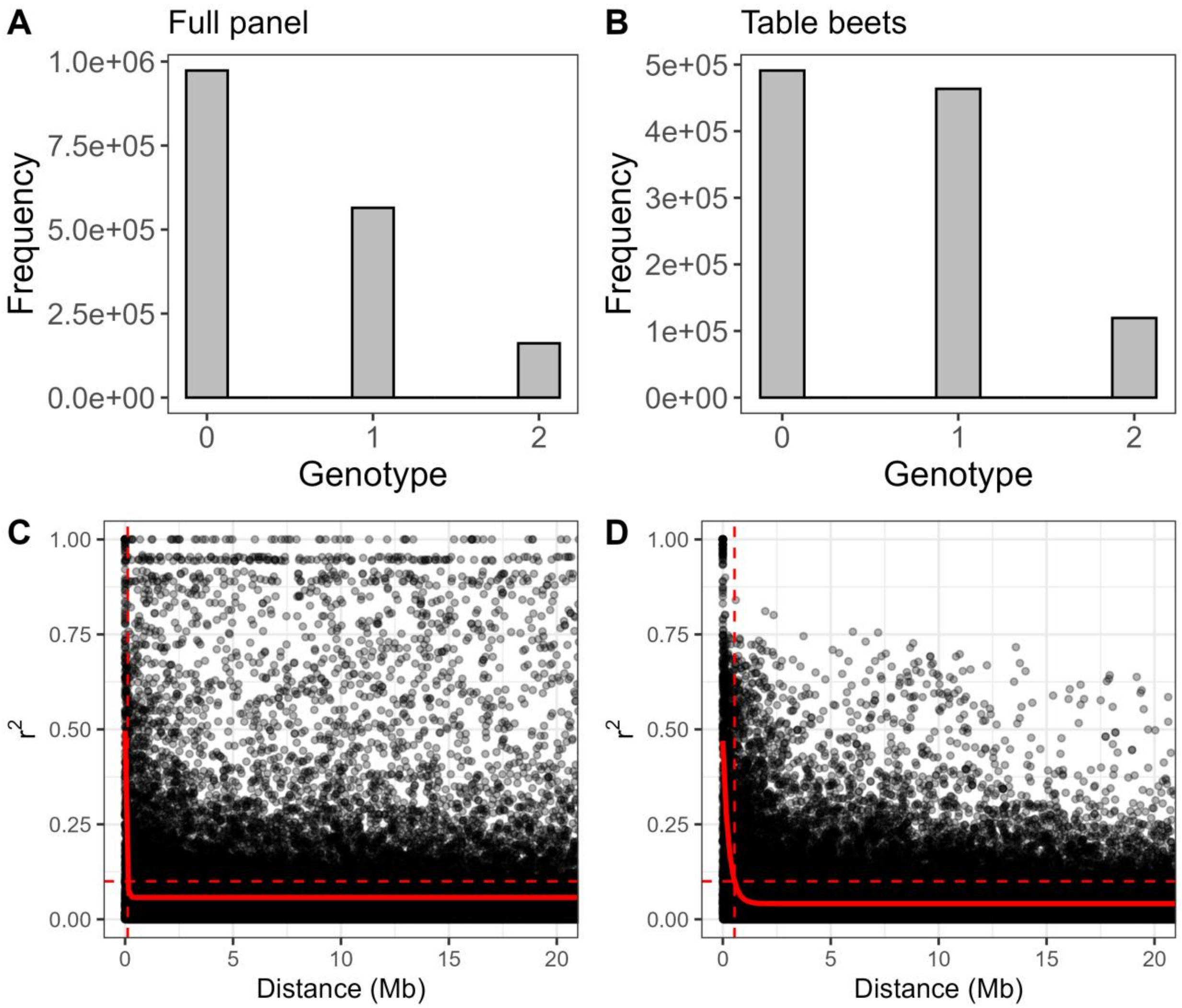

Figure S4. A-B Genotype frequencies for the (**A**) full Wisconsin Beta Diversity Panel (WBDP) and (**B**) the table beets subset. **C-D** Linkage disequilibrium (LD) decay curves are shown for (**C**) the full WBDP panel and (**D**) the table beets subset. The LD block size was determined based on the distance in megabases (Mb) at which the $r^2 = 0.1$ with values of 0.12 Mb for the full WBDP and 0.54 Mb for the table beets subset.

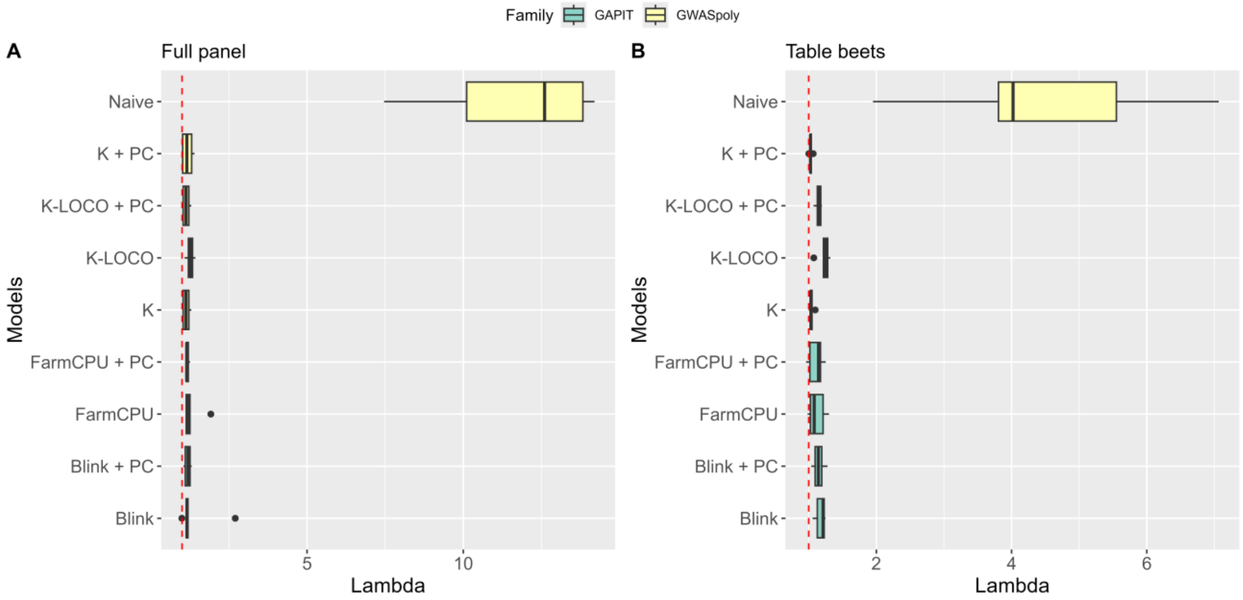

Figure S5. Effect of GWAS models and Principal Components (PC) of the genotype matrix on p-value inflation in association tests for six shape traits in (**A**) the Wisconsin Beta Diversity Panel (WBDP) and (**B**) the subset of table beets. The inflation parameter (*Lambda*) was defined as the regression slope between expected and observed $-\log_{10}$ p-values, with *Lambda* = 1 indicating no p-value inflation under the null hypothesis. Naïve indicates no population structure control; K controlled for population structure by fitting a random polygenic effect (Kinship matrix); K-LOCO used the Leave One Chromosome Out method; FarmCPU and Blink models were also tested. All models were also tested incorporating the first three principal components (+ PC). K and K-LOCO models were run in GWASpoly, while Blink and FarmCPU were run in GAPIT.

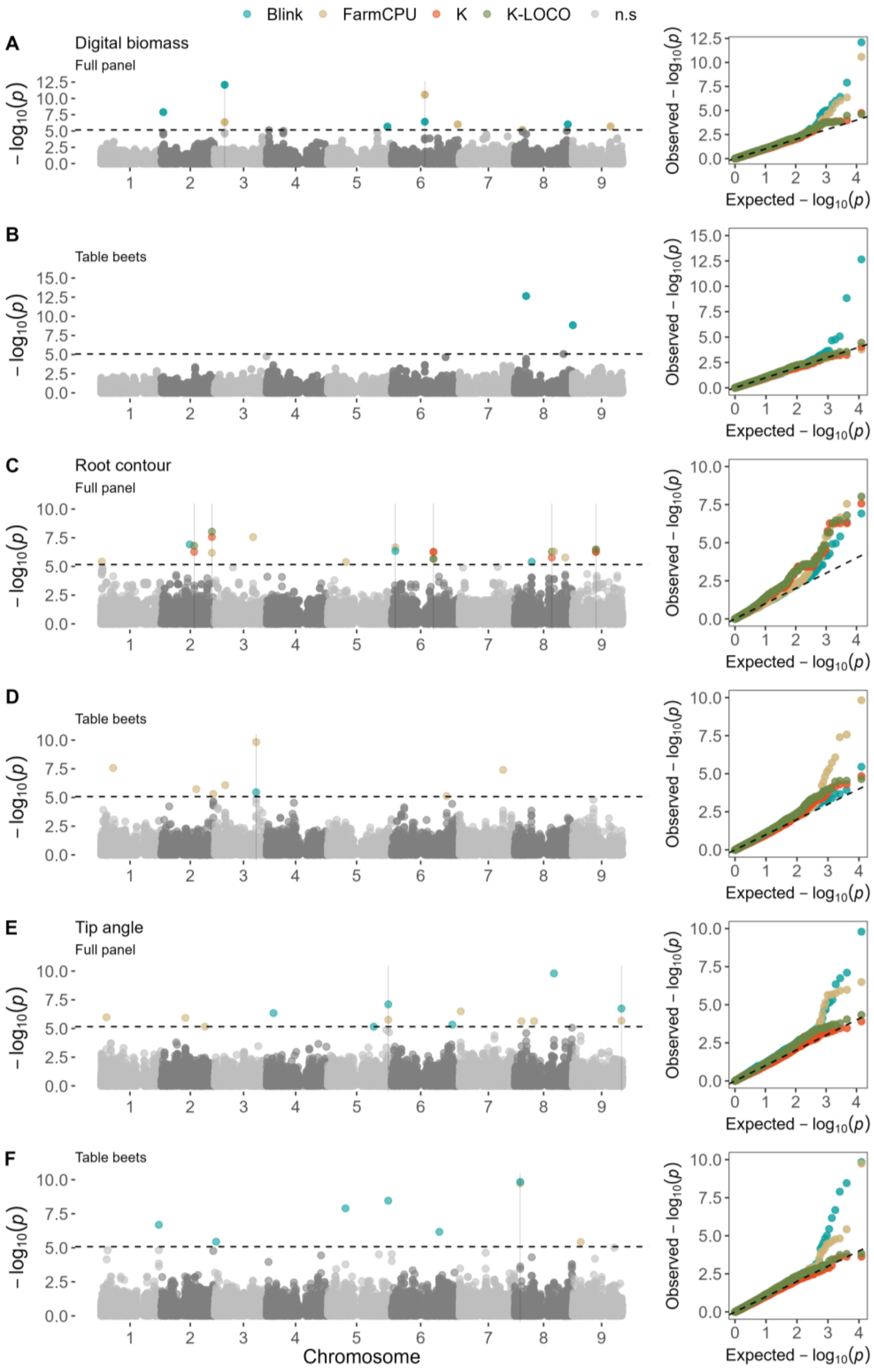

Figure S6. GWAS results for digital biomass (mm^2), root contour (PC score), and tip angle in the Wisconsin Beta Diversity Panel (WBDP) and table beet subset. **A-B** Manhattan and Quantile-Quantile (QQ) plots for the root digital biomass in (**A**) the full WBDP and (**B**) the table beet subset. **C-D** corresponding plots for root contour in (**C**) the full WBDP and (**D**) table beet subset. **E-F**

corresponding plots for tip angle in (**E**) the full WBDP and (**F**) the table beet subset. Gray vertical lines indicate QTL identified by at least two of the four statistical models tested (Blink, FarmCPU, K, and K-LOCO). The horizontal dashed line represents the Bonferroni corrected significance threshold at α=0.05. Gray points below the threshold line denote non-significant associations.

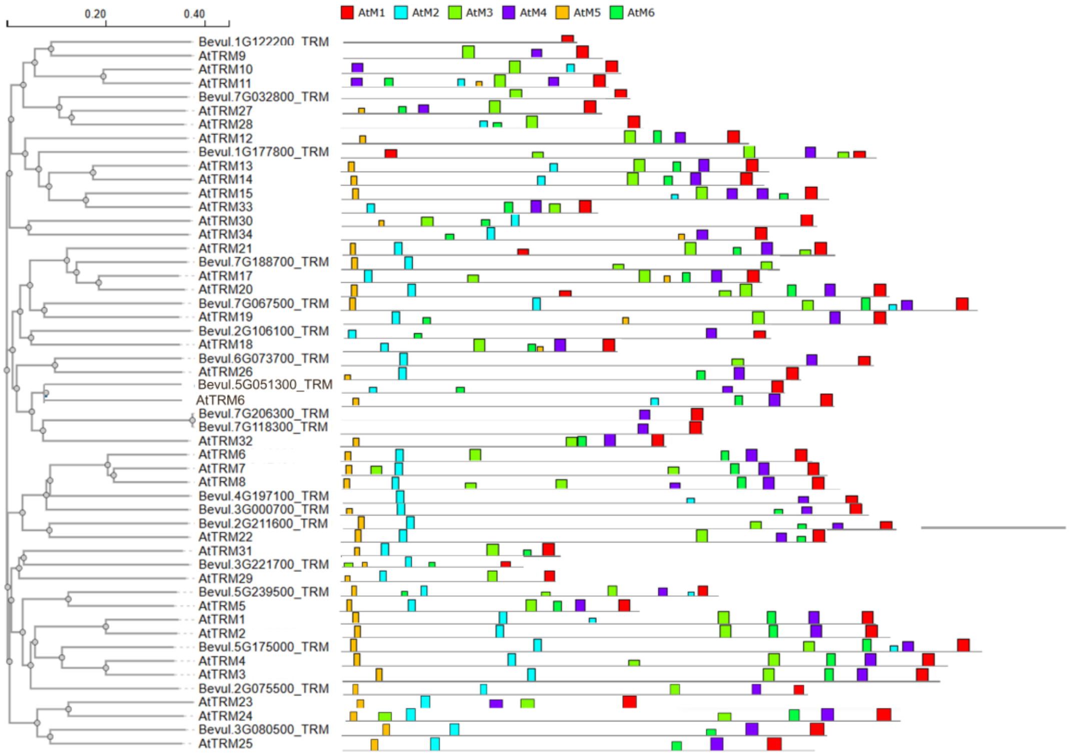

Figure S7. Phylogenetic relationships among *Beta vulgaris* and *Arabidopsis thaliana* TRM homologs. Branch lengths indicate genetic divergence, with shorter distances representing closer relationships. The motifs identified in the *Beta vulgaris* sequences using MEME are indicated by color boxes on each sequence. See Table S15 for details.

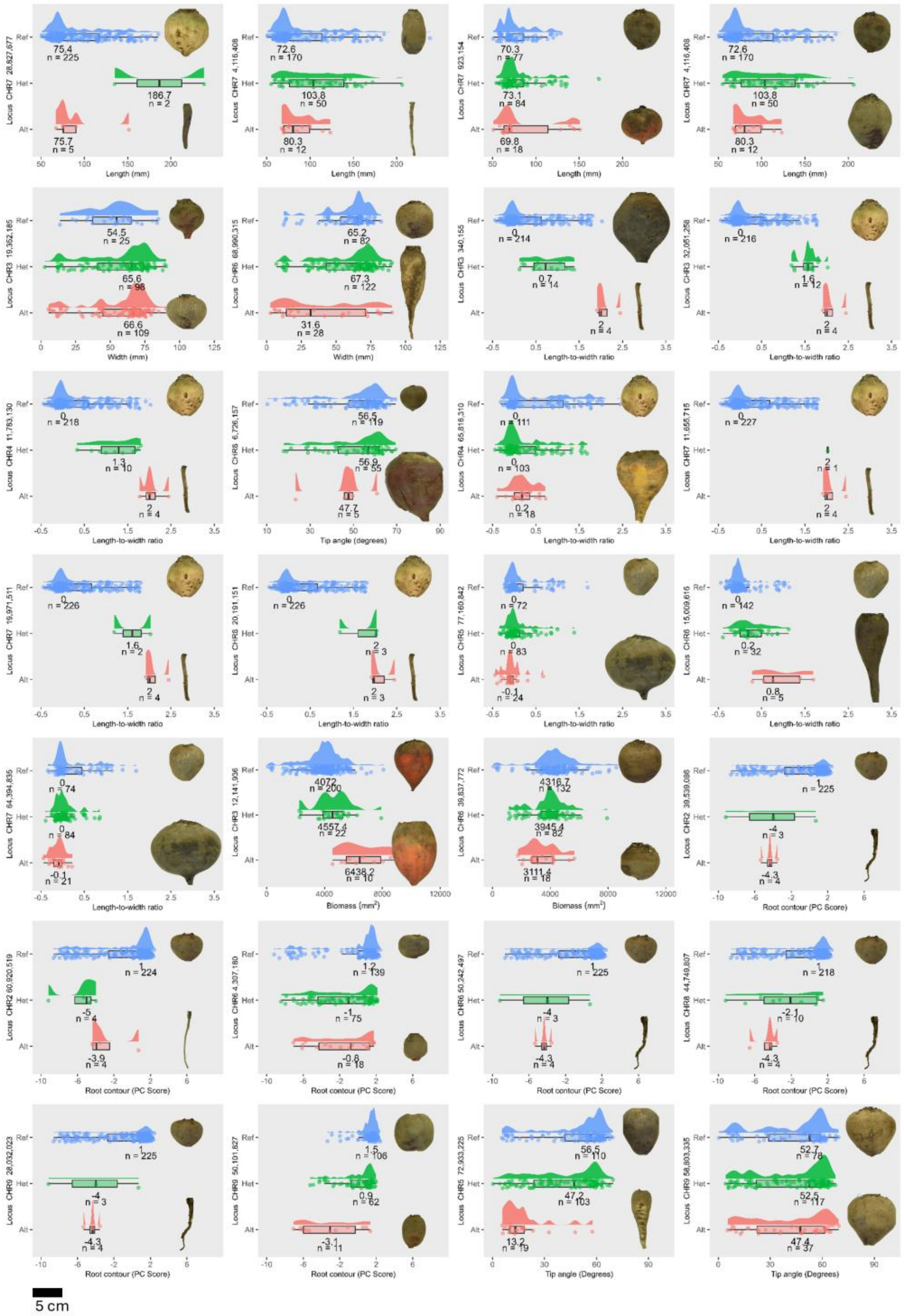

Figure S8. Effect plots show the distribution of individuals across genotypic classes (Ref: homozygous reference, Het: heterozygous, Alt: homozygous alternate) for significantly associated loci. Median trait values and sample sizes (n) are indicated for each genotypic class. Representative root images for each genotype with median scores are displayed on the right, illustrating phenotypic differences in the Ref and Alt genotypic classes. Rain and cloud plots show data dispersion and medians. Detailed locus positions are noted for each trait in the y-axis [Chromosome position (bp)]. Biomass here is digital biomass (See methods).

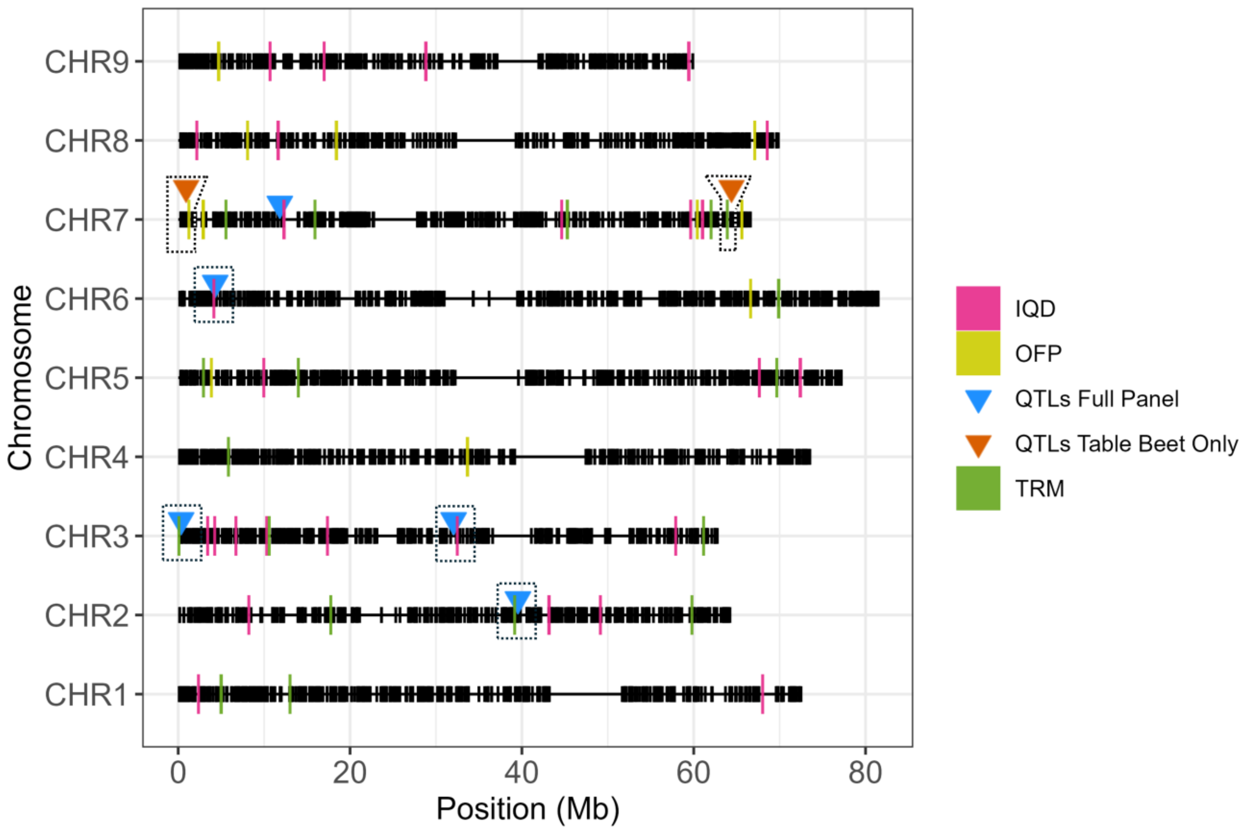

Figure S9. Approximate position of all identified members of the OFP-TRM and IQD plant shape regulon in the liftoff annotation of the table beet (*Beta vulgaris*) reference genome W357B. In boxes are significant shape-associated quantitative trait loci (QTL) (inverted triangles) less than 500kb from putative OFP-TRM-IQD regulon genes. QTL and predicted genes (vertical tiles) are color-coded by type or family. The two table beet QTL in chromosome 7 are in linkage disequilibrium with a putative OFP and TRM regulon member, respectively.

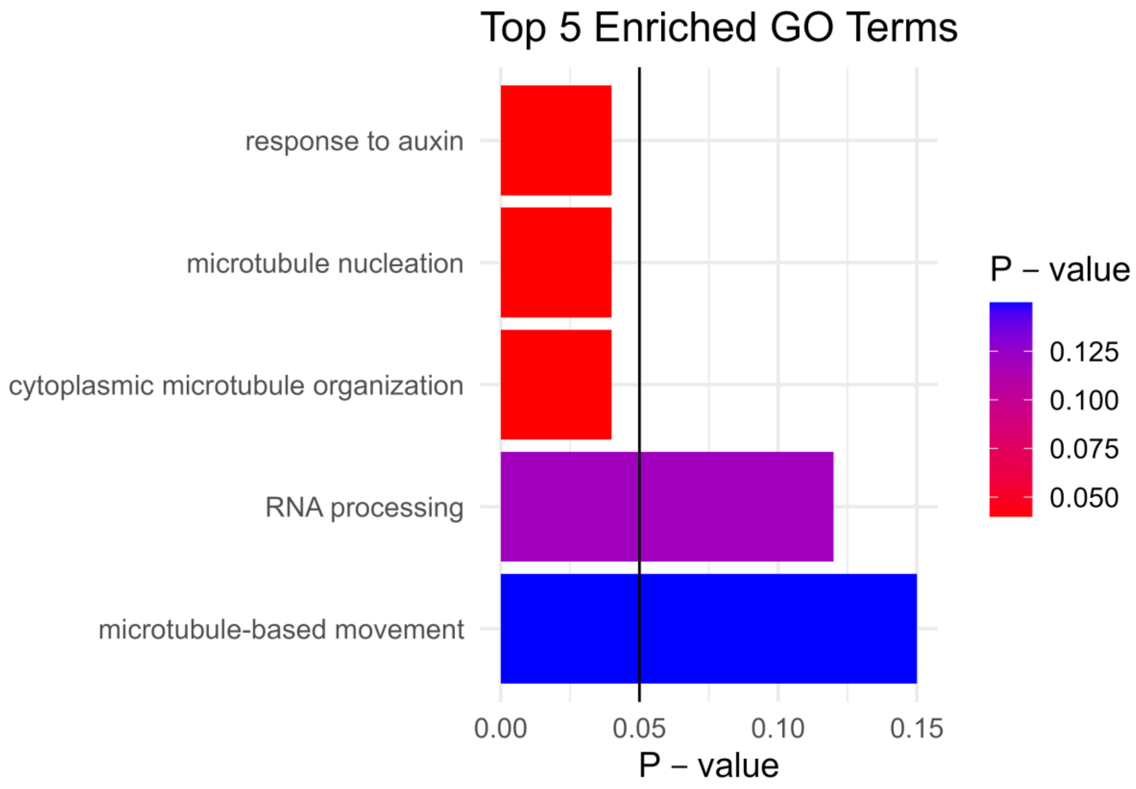

Figure S10. Top 5 enriched Gene Ontology (GO) biological process terms among target candidate genes located near significant shape-associated quantitative trait loci (QTL). GO enrichment analysis was conducted using the topGO package in R. A vertical line at p = 0.05 indicates the significance threshold.

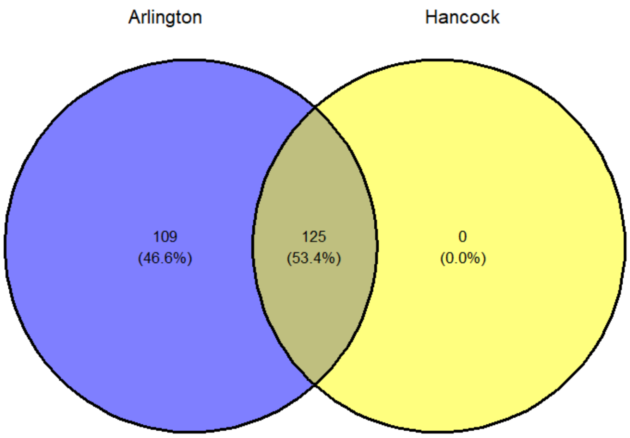

Figure S11. Distribution of planted accessions across Arlington and Hancock Agricultural Research Stations. In Arlington, a total of 234 accessions were grown in both years. A total of 109 accessions were grown in short rows plus 125 accessions grown in long rows for a total of 234. In Hancock, only 125 accessions were grown in long rows both years. The partial replication was due to lack of seed for 109 accessions.

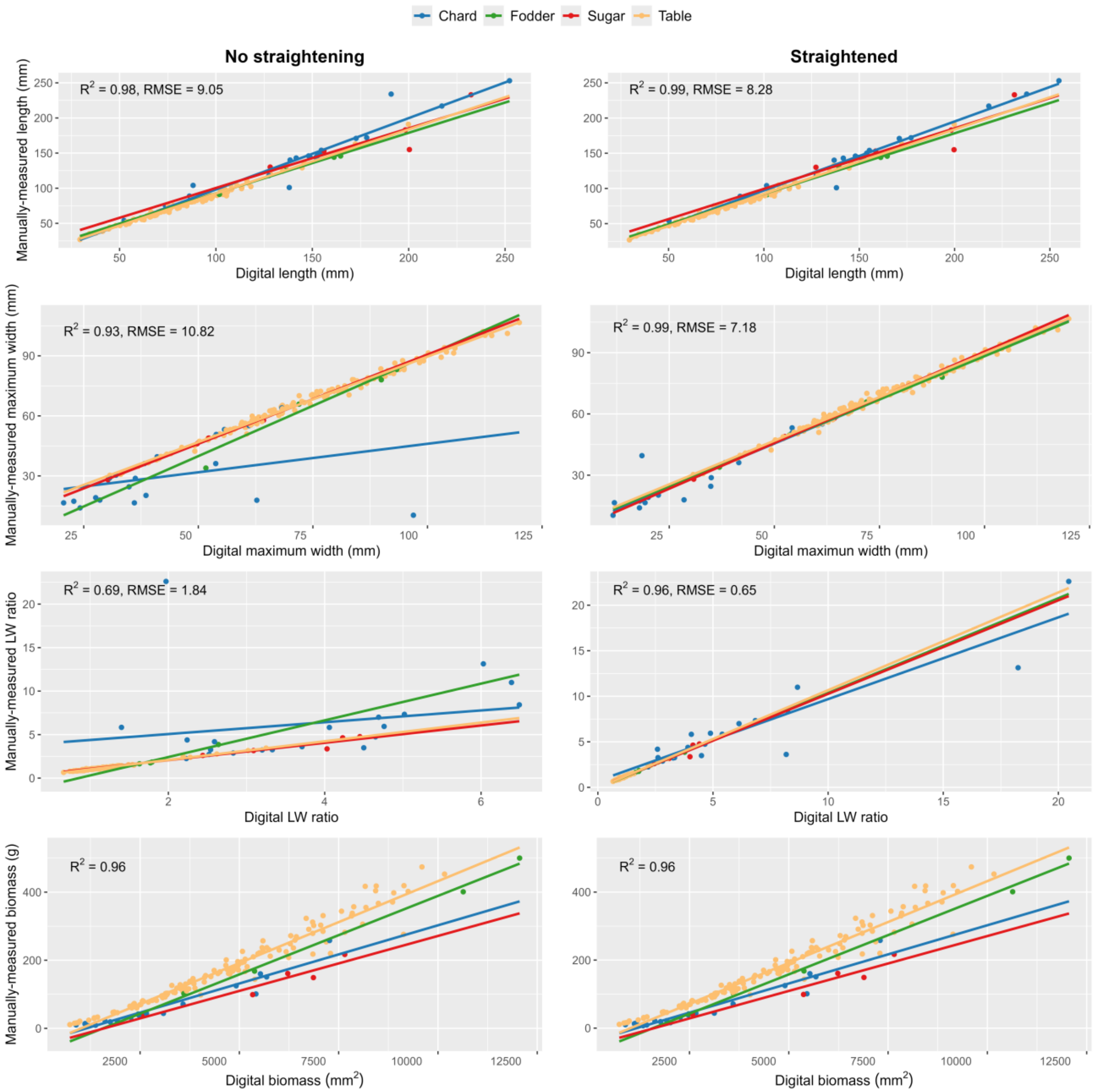

Figure S12. Validation of the digital imaging pipeline in *Beta vulgaris* subs. *vulgaris* crop complex. Manual hand-measured (y-axis) versus image-based digital (x-axis) measurements for length (mm), maximum width (mm), length-to-width-ratio, and biomass, for raw unstraightened mask (no straightening) and straightened masks for visually bent roots. Hand-measured biomass is weight in grams, while digital biomass is based on mask area (mm^2). We selected five roots from 30 accessions representing all shape groups (n=161) for validation. Pearson correlations between hand and image-based data were computed for each trait. Root Squared Mean Error (RSME) was determined for length, width, and length-to-width ratio. Straightening images (binary-masks) of visually bent roots increased correlation, lowered the RMSE across traits, and increased the accuracy of the image-based phenotype.
